# *Dictyostelium discoideum* as a Model to Assess Genome Stability Through DNA Repair

**DOI:** 10.3389/fcell.2021.752175

**Published:** 2021-10-07

**Authors:** Catherine J. Pears, Julien Brustel, Nicholas D. Lakin

**Affiliations:** Department of Biochemistry, University of Oxford, Oxford, United Kingdom

**Keywords:** *Dictyostelium*, genome stability, DNA repair, cancer, Fanconi Anemia, interstrand crosslink, PARP (poly(ADP-ribose) polymerase, *D. discoideum*

## Abstract

Preserving genome integrity through repair of DNA damage is critical for human health and defects in these pathways lead to a variety of pathologies, most notably cancer. The social amoeba *Dictyostelium discoideum* is remarkably resistant to DNA damaging agents and genome analysis reveals it contains orthologs of several DNA repair pathway components otherwise limited to vertebrates. These include the Fanconi Anemia DNA inter-strand crosslink and DNA strand break repair pathways. Loss of function of these not only results in malignancy, but also neurodegeneration, immune-deficiencies and congenital abnormalities. Additionally, *D. discoideum* displays remarkable conservations of DNA repair factors that are targets in cancer and other therapies, including poly(ADP-ribose) polymerases that are targeted to treat breast and ovarian cancers. This, taken together with the genetic tractability of *D. discoideum*, make it an attractive model to assess the mechanistic basis of DNA repair to provide novel insights into how these pathways can be targeted to treat a variety of pathologies. Here we describe progress in understanding the mechanisms of DNA repair in *D. discoideum*, and how these impact on genome stability with implications for understanding development of malignancy.

## Introduction

Maintaining genome integrity through DNA repair is critical for human health. DNA is continually being exposed to a variety of agents that induce DNA damage. This includes exogenous agents such as ultraviolet light, ionizing radiation and mutagenic chemicals, in addition to those generated during cellular metabolic processes such as reactive oxygen species or formaldehyde. These insults can lead to a wide variety of changes in DNA structure, including loss or chemical alteration of bases, chemical crosslinks (between or within a strand) and cleavage of the phosphodiester backbone to generate single (SSB) or double strand (DSB) DNA breaks. These chemically distinct lesions require a set of pathways known as the DNA damage response (DDR) to detect the different types of DNA damage and regulate mechanisms for their repair, as well as activating checkpoints to inhibit cell cycle progression. A failure of these pathways can lead to accumulation of mutations and chromosome instability that can manifest itself in a variety of pathologies, including neuro-degeneration, congenital abnormalities, immune deficiencies, and malignancy. Therefore, understanding the mechanistic basis of these DNA repair pathways provides insights into the causes of these conditions and, importantly, strategies for their treatment.

Study of DNA repair pathways by genetic approaches in a number of model organisms, most notably *Saccharomyces cerevisiae* and *Schizosaccharomyces pombe*, has been instrumental in unraveling the complexity of the DDR, identifying pathways and molecules, which are highly conserved in vertebrates. However, some of the pathways known to be critical to maintain genome stability in human cells are absent in the most commonly used models to study DNA repair. Instead, work from several laboratories over a number of years has pointed to the social amoeba *Dictyostelium discoideum* (Kessin, 2001; Pears and Gross, 2021) as an alternative model to study DNA repair mechanisms where these show limited conservation, or are difficult to study in other models (Hudson et al., 2005; Zhang et al., 2009; Couto et al., 2013b; Pears and Lakin, 2014). Intriguingly, the genome sequence of *D. discoideum* (Eichinger et al., 2005) revealed that it contains genes encoding a number of DNA repair proteins involved in resolving different types of damage that were previously believed to be restricted to vertebrates. This list includes proteins involved in repair of DNA DSBs by non-homologous end joining (NHEJ) such as DNA-PKcs and artemis (Dclre1; Block and Lees-Miller, 2005; Hudson et al., 2005; Hsu et al., 2011), in addition to XRCC1 and DNA ligase III that are involved in base excision (BER) and SSB repair (SSBR; Pears et al., 2012). In addition, *D. discoideum* has a small genome size to facilitate genetic screens and the genome often contains a limited number of orthologs of proteins, reducing the complexity caused by redundancy in mammalian systems. This is especially relevant for studying the role of histones and how their post-translational modification at the site of DNA damage regulate DNA repair. *D. discoideum* contains single copies of genes encoding the majority of histone variants (Stevense et al., 2011; Hsu et al., 2012), allowing their disruption or mutation by gene replacement or genome editing by CRISPR/Cas9 (Muramoto et al., 2010; Hsu et al., 2012; Sekine et al., 2018; Iriki et al., 2019; Asano et al., 2021). In recent years a range of methods have been developed with which to study mutation rates (Saxer et al., 2012; Pontel et al., 2016) and specific repair pathways, including NHEJ, homologous recombination (HR; Hudson et al., 2005; Couto et al., 2011, 2013a) and the post-translational modifications that regulate these pathways, such as ADP-ribosylation (ADPr; Couto et al., 2011; Kolb et al., 2018).

The presence of a wide range of repair proteins and pathways, as well as the high level of tolerance of this organism to a range of DNA damaging agents, make *D. discoideum* an attractive model to study the molecular basis of the DDR. In particular, understanding the mechanistic basis of *D. discoideum*’s resistance to DNA damage will shed light on mechanisms of intrinsic and acquired resistance of tumors to chemotherapeutic drugs that induce DNA damage. This review discusses advances made in our understanding of repair of both interstrand cross-links and DNA strand breaks in *D. discoideum* and how the study of these pathways is providing novel insights into repair mechanisms in humans. The conservation of DNA damage-induced ADPr in *D. discoideum* is then used to illustrate how *D. discoideum* can provide insights into downstream DDR mechanisms, as well as the mechanistic action of agents that target this post translational modification in treatment of cancer.

## Genome Stability and Resistance to DNA Damaging Agents

The haploid 34 Mb genome of *D. discoideum* contains around 12,000 genes arranged on 6 chromosomes along with an extrachromosomal palindrome containing the rRNA genes (Eichinger et al., 2005). The genome is AT-rich, containing 77.6% AT overall and 72% AT in coding regions. Simple sequence repeats make up an unusually high percentage (14.3%) of the *D. discoideum* genome. These are biased toward repeats of 3 and 6 nucleotides that are found in over 2000 coding regions (Eichinger et al., 2005) leading to long strings of single amino acids such as polyglutamine, that are tolerated in proteins. Genome analysis of wild *D. discoideum* populations reveal a low level of genetic variation, consistent with low mutation rates (Flowers et al., 2010), a conclusion reinforced by whole genome sequence analysis of mutation accumulation (MA) lines (McConnell et al., 2007; Saxer et al., 2012; Kucukyildirim et al., 2020). These are lines bred from a single common ancestor in which mutations generated during cell growth and division are fixed by random selection of single clonal progeny. Initial analysis of microsatellite repeats in 90 MA lines revealed low rates of mutation (McConnell et al., 2007). This was subsequently reinforced by whole genome sequence analysis of 3 and then 37 MA lines (Saxer et al., 2012; Kucukyildirim et al., 2020), revealing a mutation rate of around 2.5 × 10^–11^ base substitutions per site per generation in nuclear DNA. This is 10-100-fold lower than the majority of eukaryotic (Denver et al., 2004; Farlow et al., 2015; Long et al., 2016a; Krasovec M. et al., 2017) and prokaryotic organisms (Lee et al., 2012; Krasovec R. et al., 2017), but similar to that described in ciliates such as *Tetrahymena thermophila* (Long et al., 2016b). The GC to AT mutation rate is approximately 10-fold higher than that observed for AT to GC, which might contribute to the high AT bias in the genome. There is a two-fold higher frequency of small insertions or deletions of 1–30 bp (5 × 10^–11^ per nucleotide per generation), with the majority being small deletions (Kucukyildirim et al., 2020), compared to base substitutions. These low mutation rates are consistent with highly efficient DNA repair. However, the number of simple sequence repeats has been shown to be highly variable, both within and outside coding regions (Scala et al., 2012) consistent with a higher rate of slippage mutations (Kucukyildirim et al., 2020) and duplications of regions larger than 15 kb have been identified (Bloomfield et al., 2008).

Consistent with the low mutation rates of *D. discoideum*, vegetatively growing amoebae have long been known to be extremely resistant to a range of DNA damaging agents (Freim and Deering, 1970; Deering, 1994; Yu et al., 1998; Zhang et al., 2009), showing one of the highest levels of resistance known to ionizing radiation, which induces a variety of damage types, most notably DNA strand breaks (Deering, 1968). This high efficiency of DNA repair has been postulated to be required to counteract the damage caused on phagocytic ingestion of soil bacteria used as a food source by the amoebae (Deering, 1994). In this regard, *D. discoideum* cells disrupted in the gene encoding the DNA nuclease Xpf, which is involved in several DNA repair pathways, show a growth defect on bacteria not apparent in axenic media (Zhang et al., 2009; Pontel et al., 2016). Growth of *xpf*^–^ *D. discoideum* cells on bacteria (live or heat inactivated) led to an increased rate of mutation relative to wild type cells and to *xpf*^–^ cells grown in axenic media, as measured by accumulation of inactivating mutations in the *catA* gene, loss of which results in resistance to methanol. The mutagenic effect of phagocytosis could be due to factors generated by the bacteria, although these factors need to survive heat inactivation, or by agents generated by the *D. discoideum* amoebae to kill the ingested bacteria, such as reactive oxygen species that are known to be highly mutagenic. Interestingly, similar mechanisms are used by professional phagocytes such as neutrophils in mammals to kill ingested bacteria using a respiratory burst (Chen et al., 2007; Dunn et al., 2017). Therefore, understanding different sensitivities to growth inhibition by different bacteria in *D. discoideum* could shed light on distinct mechanisms of bacterial killing by phagocytes in the mammalian immune system.

## DNA Double Strand Break Repair and Repair Pathway Choice

The appreciation of the tolerance of amoebae to DNA damaging agents and the publication of the genome sequence triggered a resurgence in studying the mechanistic basis of the DDR in *D. discoideum*. The value of studying DSB repair in this organism (reviewed in, Pears and Lakin, 2014) was reinforced by identification of genes encoding DSB repair pathway components (Table 1) that were previously thought to be confined to vertebrates, such as DNAPKcs (Block and Lees-Miller, 2005; Hudson et al., 2005; Hsu et al., 2011). DNA DSBs are a particularly toxic form of DNA damage and cells possess a number of pathways for their repair. These include pathways based on HR in which a second copy of the DNA is used as a template for accurate repair, and pathways based on NHEJ with processing of the ends and subsequent ligation. As NHEJ can be mutagenic (for example by loss or addition of DNA during end-processing), the choice of pathway plays an important role in overall genome stability. In *D. discoideum* loss of NHEJ proteins such as Ku70/80 have a minor impact during growth on tolerance to agents that induce DSBs, presumably as HR can be employed to repair DNA damage (Hudson et al., 2005; Hsu et al., 2011). It has not been possible to generate cells deficient in core HR components, consistent with a major role for HR in genome stability in *D. discoideum*. However, disruption of the gene encoding the nuclease Exo1 leads to a decrease in HR efficiency and sensitivity to DNA DSBs during growth (Hsu et al., 2011). Exo1 is one of a number of nucleases acting in resection of the DNA DSB, a key event in HR that provides a 3′ overhang that is required for strand invasion and sequence homology searching during HR. However, further investigation revealed that, as in mammalian cells, NHEJ is active in vegetative amoebae, as evidenced by the efficiency of a plasmid insertion assay which is specific for NHEJ (Hsu et al., 2011). The delayed kinetics of DSB repair in the absence of NHEJ factors such as Dclre1 suggest that this pathway is attempted initially, but if unsuccessful then HR is employed (Hsu et al., 2011). Interestingly, *D. discoideum* undergoes a developmental life cycle in response to starvation in which it forms a multicellular structure with spores supported on a column of stalk cells. Hatching spores showed an increased dependence on NHEJ for tolerance to DSBs, suggesting that the choice to repair DSBs by NHEJ or HR may alter in some differentiated cells (Hudson et al., 2005).

**TABLE 1:** DNA repair factors experimentally verified to have a role in repair of interstrand cross links, double and single strand DNA breaks in *D. discoideum*.

DNA damage repair pathway	Protein	Gene id	Putative function	References
ICL repair	FancE FancM Ube2T FancL FancD2 FancI FancJ Xpf/FancQ Mus81 Rev3/DNApol Zeta Adprt2 APL	DDB_G0279669 DDB_G0274841 DDB_G0291199 DDB_G0292744 DDB_G0268216 DDB_G0293476 DDB_G0286621 DDB_G0284419 DDB_G0276519 DDB_G0271608 DDB_G0292820 DDB_G0293866	Bridge between core complex and FancD2 DNA-dependent helicase Ubiquitin ligase in core complex Mono-ubiquitinylated following damage, associate with damage site to recruit repair factors DNA helicase Endonuclease Nuclease Translesion DNA synthesis ADPr transferase PAR-binding protein	Zhang et al., 2009 Gunn et al., 2016
DSB repair by classical NHEJ	Adprt1a Ku70 Ku80 DNAPKcs Dclre1	DDB_G0278741 DDB_G0286069 DDB_G0286303 DDB_G0281167 DDB_G0277755	ADPr transferase DNA DSB end binding DNA DSB end binding Protein kinase forming complex with Ku78/80 heterodimer Nuclease	Couto et al., 2011 Hudson et al., 2005 Hsu et al., 2011
DSB repair by Alt-NHEJ	PolQ DNA Ligase III	DDB_G0277749 DDB_G0283857	DNA polymerase DNA ligase	Kolb et al., 2017
DSB repair by HR	Exo1 Xpf1 Rad51	DDB_G0291570 DDB_G0284419 DDB_G0273139	Exonuclease Endonuclease DNA binding and promotion of strand exchange	Hsu et al., 2011 Zhang et al., 2009 Kolb et al., 2017
SSB repair	Adprt2 Adprt1b	DB_G0292820 DDB_G0279195	ADPr transferase ADPr transferase	Couto et al., 2011

*The protein name, the gene id on dictybase (dictybase.org) and the reference demonstrating function are shown.*

So what factors regulate the choice between the NHEJ and HR pathways? An early step in NHEJ is the binding of the Ku70/80 heterodimer to DNA DSBs to initiate the pathway. Whilst disruption of the gene encoding the Ku80 subunit supresses NHEJ in vegetative *D. discoideum*, it promotes HR, indicating the binding of Ku to DSBs is a key determinant of *D. discoideum* DSB repair pathway choice (Hsu et al., 2011). This observation is consistent with the more rapid kinetics of recruitment of Ku to DSBs than HR components in mammalian cells (Kim et al., 2005) and protection of DNA ends from resection to suppress HR (Pierce et al., 2001). Indeed, it has subsequently been established that protection of DNA ends by 53BP1 and the Shieldin complex is a key regulator in promoting NHEJ at the expense of HR in human cells (Findlay et al., 2018; Gupta et al., 2018; Noordermeer et al., 2018; Setiaputra and Durocher, 2019). Whether similar mechanisms exist in *D. discoideum* remains to be tested.

## DNA Interstrand Crosslink Repair

Interstrand crosslinks (ICLs) are a toxic form of DNA damage as they prevent separation of the two strands of DNA during transcription and DNA replication. Rapidly dividing cells are therefore particularly vulnerable to ICLs. This has led to a number of drugs that induce ICLs such as platinum, cisplatin and mitomycin C, being used in chemotherapy to target a range of tumors. Therefore, a mechanistic understanding of the repair pathways used to combat ICLs will facilitate identification of genetic vulnerabilities that render tumors sensitive to these agents, and also methods to combat acquired resistance to these treatments. As well as exogenous cross-linking agents, endogenous sources of ICLs include naturally generated metabolites such as formaldehyde (Langevin et al., 2011; Nakamura and Nakamura, 2020). Vertebrates express a number of pathways to repair ICLs, including those involving the translesion DNA polymerases REV1 and REV3, and nucleases such as XPF (McHugh, 2020). The importance of ICL repair is illustrated by the developmental and health consequences of mutations in the Fanconi Anemia (FA) pathway (Duxin and Walter, 2015). FA is an inherited syndrome, with at least 22 complementation groups, that leads to progressive bone marrow failure and increased risk of various malignancies, particularly leukemia and solid tumors, as well as growth and developmental defects, including skeletal abnormalities and short stature. Cells from FA patients show increased sensitivity to ICL-inducing agents such as cisplatin and FA proteins are involved in repair of ICLs, particularly when replication forks encounter these lesions during S-phase. The identification of formaldehydes as the predominant source of damage repaired by the FA pathway also raises the possibility that it is of particular importance for the type of cross-links induced by this agent, including cross-linking DNA to protein (Langevin et al., 2011). In the vertebrate FA pathway, a large core complex of 11 or more proteins associates with chromatin upon DNA damage in which the catalytic subunit FANCL functions as an E3 ubiquitin ligase to mono-ubiquitinylate both FANCI and FANCD2. These form a heterodimeric complex, and their ubiquitylation is required for tolerance to ICL by integrating nucleotide excision repair (NER), a pathway also used to remove UV-induced thymidine dimers, with translesion DNA polymerases and HR to restore genome integrity (Garcia-de-Teresa et al., 2020).

The number of pathways to repair ICLs has expanded with organism complexity. In *S. cerevisiae* repair of ICLs is mainly due to NER (Lehoczky et al., 2007). Whilst the majority of proteins associated with FA are not conserved in yeast, the *D. discoideum* genome contains an expanded repertoire of FA proteins similar to those observed in humans (Zhang et al., 2009). This includes orthologs of genes encoding the heterodimer FANCI/D2 and the E2 (Ube2T) and E3 (FANCL) ubiquitin ligases required for their modification (Table 1). However, the majority of core complex components are not apparent by sequence homology, suggesting a simplified version of the pathway may operate. *D. discoideum* strains deficient in the *fanc* genes such as *fancD2* or *fancL* indeed show mildly increased sensitivity to cisplatin, as do strains deficient in the translesion DNA polymerase *rev3*. However, the majority of *D. discoideum*’s tolerance to cisplatin requires the nuclease Xpf, as *xpf*^–^ cells show 50–100 fold greater sensitivity to cisplatin than cells disrupted in *fancD2* or *rev3*. Double mutants, such as *xpf^–^fancD2^–^* strains, do not show additive sensitivity. This suggests that Xpf does function in the *D. discoideum* FA pathway, but that its major role in tolerance to cisplatin is independent of this pathway. This alternate role for Xpf is not in NER as disruption of another component of this pathway does not sensitize *D. discoideum* cells to cisplatin, but could be related to a role in HR. Subsequently, mutations in *xpf* have been found in FA patients (Bogliolo et al., 2013; Kashiyama et al., 2013) and studies in mice and human cells demonstrate that the role for XPF in tolerance to ICL involves a pathway other than its known roles in excision repair (Mulderrig and Garaycoechea, 2020).

*Dictyostelium discoideum* offers an easily tractable system to unravel the complex interactions between repair pathways which may depend on the exact nature of the ICL, and phase of the cell cycle. In this respect it is noteworthy that *D. discoideum* amoebae are predominantly in G2 phase of the cell cycle, with a very short or undetectable G1 phase (Durston et al., 1984; Weijer et al., 1984), offering one potential explanation for the minimal requirement of the FA pathway for tolerance to cisplatin. In addition, cisplatin favors GC crosslinks and it will also be of interest to compare the involvement of different pathways using agents that induce other forms of cross-link that may be more abundant in the AT-rich *D. discoideum* genome. In particular the developmental defects in FA patients are believed to be due to endogenous metabolites that are able to form crosslinks such as formaldehyde (Langevin et al., 2011). However, the sensitivity of *D. discoideum* cells deficient in different pathway components to such agents is not known.

In a parallel approach, identification of mutant *D. discoideum* strains that are resistant to cisplatin has revealed sphingolipid metabolism as a regulator of tolerance to this chemotherapeutic drug (Min et al., 2004, 2005a; Alexander et al., 2006). For example, overexpression of sphingosine-1-phosphate lyase or loss of sphingosine kinase, both of which lead to a reduction in levels of sphingosine-1-phosphate, results in increased sensitivity to cisplatin (Min et al., 2004). This was subsequently shown to also be true in human cells, providing pharmacological targets for increasing tumor sensitivity to cisplatin (Min et al., 2005b). Gene expression studies revealed genes with expression levels altered on cisplatin treatment. Many of these changes were altered in resistant mutants and subsequent disruption of five of these also altered cisplatin sensitivity, revealing novel tolerance pathways (Van Driessche et al., 2007).

## Strand Break Repair and ADP-Ribosylation

**FIGURE 1:**
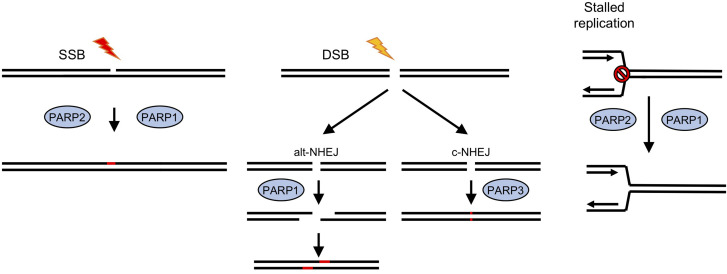
Roles for ADP-ribosyl transferases in strand break repair in human cells. In human cells PARPS have a well-established role in DNA repair (Caldecott, 2014; Azarm and Smith, 2020). PARPs 1 and 2 have been implicated in repair of single strand breaks (SSB), which are induced either directly by exogenous or endogenous damaging agents, or as a result of base excision repair (BER) pathways following base damage. Recruitment of PARPs1/2 to the break site and subsequent ADP-ribosylation of proteins, including histones at the damage site, lead to recruitment or stabilization of repair factors and subsequent damage resolution. DNA double strand break (DSB) repair by classical non-homologous end joining (c-NHEJ) involves a third PARP, PARP3, whereas an alternative version (alt-NHEJ) is dependent on PARP1. Stalled replication forks can lead to fork collapse and restart is also thought to be PARP-dependent.

One family of DNA repair enzymes that are found in *D. discoideum* that are absent or show limited conservation in other non-vertebrates are ADP-ribosyl transferases (ARTs), a family of proteins that catalyze the addition of single or poly-ADP-ribose moieties onto target proteins using NAD^+^ as substrate (Gibson and Kraus, 2012; Vyas et al., 2014; Martin-Hernandez et al., 2017). In humans, 17 genes containing predicted ART domains have been identified (Hottiger et al., 2010). ADPr has been implicated in a wide variety of processes, including cell growth and differentiation, transcriptional regulation and programmed cell death (Gibson and Kraus, 2012) and in a number of diseases, including cancer, neurological disorders, and cardiovascular disease (Palazzo et al., 2019). However, the best-defined role of these enzymes, which are also known as poly ADPr-polymerases (PARP)s, is in regulating DNA repair, particularly of DNA strand breaks (Figure 1; Caldecott, 2014; Azarm and Smith, 2020). PARP1, the founder member of the PARP family in mammalian cells, recognizes SSBs generated directly or as a consequence of base excision repair (BER), and becomes activated to ADPr substrates at the break site. This promotes accumulation of the XRCC1 scaffolding protein at DNA damage sites that subsequently leads to assembly of repair factors at the lesion that resolve the damage (Caldecott, 2014). Small molecule inhibitors of PARPs are in use in the clinic to treat cancers which are deficient in HR, most notably those with mutations in *BRCA1* and *BRCA2*. Loss of function of these genes predisposes individuals to developing breast and ovarian cancers (Bryant et al., 2005; Farmer et al., 2005; Slade, 2020). Therefore, understanding the mechanistic basis of how PARPs regulate repair of different DNA lesions and how redundancy between these pathways allows cells to tolerate DNA damage will facilitate identification of cancers responding to PARP inhibition and suggest mechanisms to overcome acquired resistance to these agents.

**FIGURE 2:**
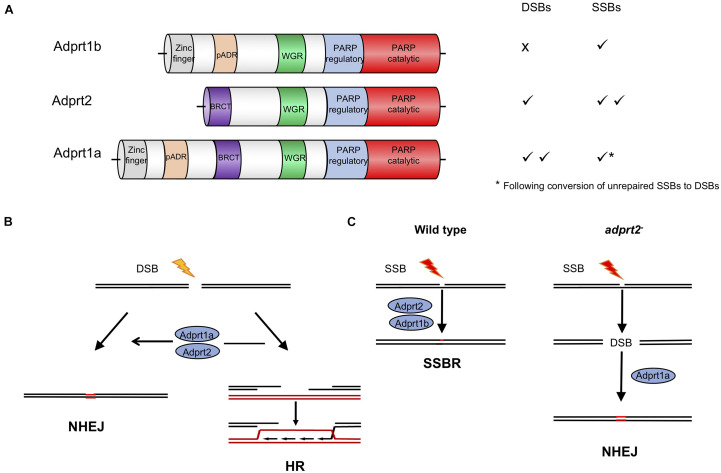
Roles for ADP-ribosyl transferases in strand break repair in *D. discoideum* cells. **(A)** Three *D. discoideum* ARTS have been demonstrated to have a role in DNA repair of strand breaks. As well as a catalytic domain at the C terminus, all three contain N terminal motifs associated with DNA repair proteins in mammals, including BRCT, WGR and zinc finger domains (Otto et al., 2005; Citarelli et al., 2010; Pears et al., 2012). **(B)** Adprt1a and, in its absence Adprt2, regulate choice between repair of DSBs by NHEJ or HR. Their action favors NHEJ by stabilizing recruitment of the Ku70/80 heterodimer to the ends, protecting them from end resection by nucleases, the initial step in the HR pathway. In *D. discoideum* Ku70 contains a PBZ domain, an ADPR-binding motif, required to stabilize its association with damaged chromatin (Couto et al., 2011; Hsu et al., 2011). **(C)** In wild type cells Adprt2 and Adprt1b are required for tolerance to SSBs. In *adprt2*^–^ cells, unrepaired SSBs can be converted to DSBs and repair is then dependent on Adprt1a and subsequent Ku70/80 recruitment to promote NHEJ (Couto et al., 2011, 2013b).

Poly ADPr-polymerase activity was identified in *D. discoideum* many years ago (Rickwood and Osman, 1979; Kofler et al., 1993; Rajawat et al., 2007; Mir et al., 2015) and a cDNA sequence revealed considerable homology to mammalian PARPs (Auer et al., 1995). The completed genome sequence of *D. discoideum* (Eichinger et al., 2005) revealed 15 genes encoding proteins with putative ART catalytic domains. Four of these proteins also contain other domains found in human PARPs that respond to DNA damage, including protein-protein interaction domains such as BRCT domains, and nucleic acid binding WGR and zinc fingers domains (Figure 2A; Otto et al., 2005; Citarelli et al., 2010; Couto et al., 2011; Pears et al., 2012). As in humans, two PARPs (Adprt1b and 2) play a role in SSBR/BER as their loss leads to sensitivity to hydrogen peroxide and methanesulphonate (MMS), agents that induce damage that requires these pathways for repair (Couto et al., 2011). However, a third ART, Adprt1a, that is not required for tolerance to DNA SSBs, plays a role in DNA DSB repair (Couto et al., 2011). Importantly, disruption of the *adprt1a* gene results in a reduced ability to retain Ku70 at DSBs, with a consequent reduction in NHEJ efficiency (Figure 2B; Couto et al., 2011). Studies carried out at a similar time also revealed a role for a third ART, PARP3, in promoting NHEJ in human cells (Boehler et al., 2011; Rulten et al., 2011; Beck et al., 2014), confirming the value of the *D. discoideum* model.

In a pre-genome editing era, an important strength of the *D. discoideum* model was the ability to disrupt multiple genes in combination to assess genetic interactions and redundancies within biological pathways. This approach highlighted novel compensatory repair mechanisms that are employed upon inactivation of PARPs, particularly with reference to *D. discoideum* cells lacking Adprt2, the major ART required for tolerance to DNA SSBs (Figure 2C). Intriguingly, *adprt2*^–^ cells still show robust ADPr of proteins following treatment with MMS which induces DNA base alkylation that is subsequently converted to SSBs during BER. However, this is lost on further deletion of the gene encoding the DSB-responsive ART, Adprt1a (Couto et al., 2013b) and *adprt1a*^–^ *adprt2*^–^ double mutant cells show increased sensitivity to MMS relative to cells with disruption of the *adprt2* gene alone. The known role for Adprt1a in NHEJ suggested that in the absence of effective repair, SSBs are converted to DSBs that are subsequently repaired by NHEJ. Consistent with this hypothesis, *adprt2*^–^ cells show increased recruitment of Ku80 to chromatin following MMS treatment that is dependent on the presence of Adprt1a. Moreover, disruption of NHEJ pathway components in *adprt2*^–^ cells increases the sensitivity of these cells to MMS. Together, this analysis reveals the importance for NHEJ in the absence of effective SSB repair. Therefore redundancy between PARP-dependent repair mechanisms is not only due to multiple PARPs regulating the same repair mechanism, but also disruption of one specific PARP-dependent repair pathway channeling DNA damage through alternate repair routes that are dependent on another PARP. Importantly, these studies prompted analysis of similar functional overlap of human PARPs in regulating DNA repair. It has been known for some time that both PARP1 and PARP2 function in repair of DNA SSBs (Schreiber et al., 2002; Menissier de Murcia et al., 2003; Hanzlikova et al., 2017). However, more recently it has also become apparent that, similar to *D. discoideum*, disruption of SSB repair can channel DNA damage through alternate repair mechanisms that employ other PARPs (Ronson et al., 2018). This redundancy has implications for use of inhibitors specific for individual PARPs, suggesting that inhibitors targeting several PARPs involved in distinct DNA repair pathways may be required for complete loss of repair functions in chemotherapy. Understanding these relationships in organisms such as *D. discoideum* will provide important information about redundant PARP-dependent repair mechanisms in human cells and potential clinical applications.

## Identifying ADP-Ribosylation Binding Motifs in *D. discoideum* to Expand the Role for ADP-Ribosylation – a Novel Role for ADP-Ribosylation in Repair of Interstrand Crosslinks

Once activated, ARTs catalyze ADPr of proteins at sites of DNA damage leading in turn to recruitment of DNA repair and chromatin remodeling factors to aid resolution of the damage. This is achieved by interaction between proteins containing PAR-binding motifs with mono- or poly-ADP-ribosylated proteins. A number of these PAR binding motifs (PBM) have been identified, including a 20 amino-acid PBM, PAR-binding zinc finger (PBZ), WWE, and macro-domains (Gibson and Kraus, 2012). Bioinformatic identification of proteins containing these motifs is an invaluable tool to identify downstream response systems. Three PBZ domain containing proteins have been identified in vertebrates, CHFR, APLF, and APL (Ahel et al., 2008). All of these proteins have been implicated in the DDR, and the PBZ domains of APLF and CHFR are required for association of these proteins with sites of DNA damage. *D. discoideum* contains an expanded family of seven PBZ-domain containing proteins (Ahel et al., 2008), implicating ARTs in the regulation of these proteins, and the pathways in which they function. For example, *D. discoideum* Ku70 contains a PBZ domain which is required for efficient enrichment of the Ku70/Ku80 complex on chromatin in response to DSBs and efficient NHEJ (Couto et al., 2011). Interestingly, vertebrate Ku70 does not contain a PBZ domain, although NHEJ is dependent on PARP activity in these organisms (Boehler et al., 2011; Rulten et al., 2011; Beck et al., 2014). This suggests that different mechanisms for recruitment of NHEJ proteins may have arisen during evolution, and that the expanded number of ADPr-binding domains in *D. discoideum* may provide a novel approach to identify response systems downstream of ADPr.

Macrodomains are more diverse, binding a range of motifs such as MAR, PAR and O-acetyl ADP-ribose and a number have been characterized as domains that catalyze the metabolism of ADPr such as PAR and MAR hydrolysis to remove these modifications (Rack et al., 2016, 2020). Again in vertebrates a number of proteins with a role in the DDR (Jankevicius et al., 2013; Rosenthal et al., 2013; Barkauskaite et al., 2015) contain macro domains which are required for their recruitment to site of damage, including ALC1 and the histone variant macroH2A1.1 (Ahel et al., 2009; Gottschalk et al., 2009; Timinszky et al., 2009). Bioinformatic analysis identified six *D. discoideum* proteins containing macrodomains (Gunn et al., 2016). As proof of concept, this list contained DNA ligase III, a DNA repair protein that does not contain a macrodomain in vertebrates. A novel protein identified in this approach, APL, contains an unusual macrodomain with a circular permutation which interacts with PAR in an *in vitro* binding assay. Interestingly, APL also contains a PBZ domain with PAR binding activity. One interpretation of this is that these two domains may act in tandem to increase binding affinity to a PAR chain, or that APL may bridge ADPr proteins to generate a complex. The presence of another protein-protein interaction FHA domain and the lack of any domain containing recognized enzymatic activity would be consistent with a scaffolding role for APL.

The identification of APL in *D. discoideum* revealed a novel role for ADPr in tolerance to cisplatin (Table 1). APL enrichment to chromatin was only detectable following treatment with cisplatin, and not with agents inducing predominantly SSBs or DSBs, the classical pathways that depend on ADPr (Gunn et al., 2016). Deletion of the macrodomain alone severely abrogated APL recruitment to chromatin following induction of ICLs, despite the presence of the intact PBZ and FHA domains, and this event was largely dependent on Adprt2, an enzyme previously implicated in *D. discoideum* SSB repair. A role for ADPr in ICL repair has subsequently been demonstrated in human cells (Li et al., 2020). ADPr by PARP1 is required to recruit the NEIL3 DNA glycosylase to ICLs and loss of either NEIL3 or inhibition of ADPr leads to increased sensitivity to ICLs, in a pathway distinct from the FA pathway. This example demonstrates the power of using the presence of ADPr-binding domains as surrogate markers for novel PARP-dependent pathways and highlights the importance of characterizing other *D. discoideum* proteins containing PBZ and macro domains in the future (Ahel et al., 2008; Gunn et al., 2016).

## Targeting of ADP-Ribosyl Transferases in Cancer

**FIGURE 3:**
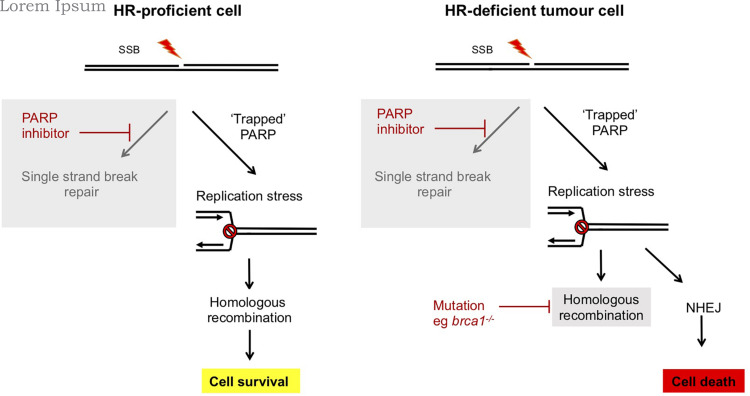
Synthetic lethality of PARP inhibitors in treatment of breast and ovarian cancers deficient in BRCA1/2. In normal human cells, single strand breaks are repaired by PARP-dependent SSBR. In the presence of ART inhibitors PARP is trapped at the lesion and this impedes DNA replication. However, this replication stress can be repaired by an HR dependent mechanism and cells survive. However, in tumor cells which are deficient in components of HR, such as *brca1/2^–/–^* breast or ovarian cancers, HR-dependent resolution of replication stress is not possible and instead mutagenic NHEJ pathways prevail. This results in selective killing of tumor cells by PARP inhibitors (Bryant et al., 2005; Farmer et al., 2005; Slade, 2020).

Redundancy between repair pathways in tolerance of DNA damage has been exploited in a synthetic lethal strategy to selectively kill cancer cells which are deficient in repair pathways. This deficiency likely promotes the accumulation of mutations driving tumor formation, but can also provide an Achille’s heel to target in treatments (Bryant et al., 2005; Farmer et al., 2005; Slade, 2020). PARP inhibitors function by trapping PARPs at DNA SSBs, leading to a block in DNA replication and fork collapse (Rouleau et al., 2010). This generates DNA DSBs that under normal circumstances can be repaired by HR in a pathway that is dependent on BRCA1/2. Therefore, in the absence of these, or other HR factors, PARP inhibitor-induced DSBs are repaired by mutagenic DNA repair pathways (both classical c-NHEJ and atypical alt-NHEJ) that results in genome instability and cell death (Figure 3). Therefore, PARP inhibitors are selectively toxic to HR-deficient tumor cells while HR proficient non-tumor cells remain viable. PARP inhibitors such as Olaparib are in use in the clinic to treat breast and ovarian cancers that are deficient in BRCA1 or BRCA2, encoded by genes associated with inherited susceptibility to breast cancer (Slade, 2020).

Treatment of *D. discoideum* with the PARP inhibitor NU1025 leads to chromatin recruitment of the HR protein Rad51, indicating that, similar to humans, DNA damage induced by PARP inhibitors is repaired by HR. Consistent with this model, strains lacking the nuclease Exo1 that are defective in HR, show synthetic lethality with PARP inhibitors (Kolb et al., 2017). The conservation of this phenomenon with humans makes *D. discoideum* an attractive system to identify mutations which lead to resistance of HR-deficient strains to PARP inhibitors and therefore potentially cause resistance of tumors to PARP inhibitor treatment. In human cells two pathways have been proposed to compete with HR to repair DNA damage: c-NHEJ and alt-NHEJ (Ceccaldi et al., 2015; Mateos-Gomez et al., 2015). Disruption the NHEJ gene *dnapkcs*, supresses sensitivity of *D. discoideum exo1*^–^ cells to PARP inhibitors. This is consistent with mutagenic c-NHEJ becoming the predominant pathway to repair PARP inhibitor-induced DNA damage and driving inhibitor toxicity when HR is absent. In contrast, disruption of genes encoding DNA ligase III and DNA polymerase theta, components of alt-NHEJ (McVey and Lee, 2008), does not impact on tolerance to PARP inhibitors either in the presence or absence of Exo1. This could reflect the dependence of alt-NHEJ on PARP. The specificity of involvement of distinct ARTs in different pathways highlights the desirability of unraveling their specific roles and pathway interactions, and for developing PARP inhibitors that are specific for different ARTs in the clinical setting. Currently, clinically approved PARP inhibitors such as Olaparib are broad spectrum inhibitors with activity toward all known ARTs involved in DNA repair, but inhibitors specific for individual ARTs are under development (Slade, 2020).

Understanding the interplay between PARP-dependent and -independent repair mechanisms will aid identification of tumors that are likely to respond to PARP inhibitors either alone, or in combination therapies, in addition to mechanisms by which tumors become resistant to these agents. For example, reactivation of HR in mammalian cells by loss of the DNA end resection inhibiting factor 53BP1 can cause resistance to PARP inhibitors (Cao et al., 2009) and has been reported in BRCA-deficient breast cancers (Bouwman et al., 2010). Screens to identify 53BP1 effectors identified 53BP1-interacting proteins as components of the shieldin complex (Findlay et al., 2018; Gupta et al., 2018; Noordermeer et al., 2018), which acts to protect DNA ends from end resection (Setiaputra and Durocher, 2019). The ability to rapidly generate strains with multiple gene disruptions in *D. discoideum* facilitates analysis of this druggable pathway as resistance can be easily investigated following blocking combinations of pathways in the presence or absence of inhibitors. For example, this approach demonstrated that resistance to PARP inhibitors observed by disrupting NHEJ in an *exo1*^–^ background (*dnapkcs^–^exo1^–^* cells) is mediated by promoting end-resection to reactivate HR. Small molecule inhibitors of Mre11, a second nuclease involved in strand resection during HR, restores sensitivity of *dnapkcs^–^exo1^–^* cells to PARP inhibitors (Kolb et al., 2017). This highlights redundancy between the nucleases Exo1 and Mre11 in HR and suggests combination approaches to avoid resistance in cells deficient in one nuclease.

The importance of understanding the role for ADPr in interplay between repair pathways in cancer therapy is also apparent when considering repair of ICLs by drugs such as cisplatin, which are used to treat a number of cancers. Studies in *D. discoideum* first revealed a role for ADPr in tolerance to cisplatin and, as initially demonstrated for SSBR, inhibition of one ART leads to channeling the damage to a second ART-dependent repair pathway (Gunn et al., 2016) reminiscent of the interplay identified for SSB repair (Couto et al., 2013b). *D. discoideum* cells lacking Adprt2 (the major ART required for SSB repair) show increased sensitivity to cisplatin. However, in the absence of Adprt2, the damage is channeled into an Adprt1a-dependent pathway, which is most likely NHEJ. In human cells, a PARP-dependent pathway has been recently identified as playing an important role in repair of ICL (Li et al., 2020). The consequence of disrupting genes encoding NHEJ proteins in cells deficient in ICLs repair, such as FA cells, has been found to lead to either increased or decreased tolerance to cisplatin (Houghtaling et al., 2005; Adamo et al., 2010; Pace et al., 2010; Bunting et al., 2012). This difference may reflect the cell cycle profile of the cells. *D. discoideum* cells are predominantly in G2 phase (Weijer et al., 1984) and these may utilize NHEJ effectively whereas NHEJ is particularly toxic when used during S phase (Rothkamm et al., 2003). Overall understanding the interplay between ARTs in repair of different types of DNA damage and the generation of inhibitors specific for particular ARTs will allow for improved treatment strategies.

## Future Directions

*Dictyostelium discoideum* clearly has much to offer in terms of a system to study the DDR both to delineate pathway mechanisms and investigate the interplay between pathways that play a vital role in genome stability. Initial interest focused on genetic analysis of strains disrupted in pathway components shared with mammalian cells, though the development of genome editing techniques means this analysis, in addition to genome-wide genetic screens, is now feasible in human cells. However, *D. discoideum* still has an important role to play in this regard, for example in instances where human cells contain multiple copies of genes encoding enzymes with overlapping activities. An important example of this is probing modification of histone proteins as the *D. discoideum* genome has mostly single copy genes encoding a range of histone variants (Stevense et al., 2011; Hsu et al., 2012). Introduction of mutations into endogenous single copy histone genes has been used to demonstrate a role for these in transcriptional memory, interplay between histone modifications, and in tolerance of cells to agents that target histone deacetylases used in cancer treatments (Muramoto et al., 2010; Hsu et al., 2012; Huang et al., 2021). It has long been apparent that histones are a major substrate for ADPr following DNA damage, and this is also true in *D. discoideum* (Rickwood and Osman, 1979; Rajawat et al., 2007; Couto et al., 2011; Rakhimova et al., 2017). Advances in mass spectrometry have recently revealed the major sites on histones that are ADPr in response to DNA damage (Jungmichel et al., 2013; Hottiger, 2015; Leidecker et al., 2016; Palazzo et al., 2018; Chen et al., 2021). The ability to manipulate multiple genes simultaneously and, in particular, to introduce mutations into endogenous histone proteins both singly and in combination (Rakhimova et al., 2017; Asano et al., 2021), offers a unique opportunity to study the role of these modifications in orchestrating DNA repair pathways.

Whilst screens of mutations introduced by CRISPR/Cas9 are now possible in human cells, the mutations are loss of function and the large number of genes, often with redundant functions, make these screens time consuming and expensive. The small genome size of *D. discoideum* facilitates saturation screens following treatment with DNA damaging agents and their subsequent identification by whole genome sequencing. Importantly, this approach has been shown to identify dominant, gain of function, as well as recessive mutations (Li et al., 2016). Therefore, *D. discoideum* has important potential in screens for example for tolerance to DNA damaging agents used in chemotherapy or mutations that drive resistance to these and other agents, for example PARP inhibitors. The ability to specifically manipulate genes offers the further possibility to perform these experiments in a variety of genetic backgrounds such as those containing mutations found in human cancer predispositions. In an alternative approach, genes encoding a small number of proteins implicated in DNA repair pathways in other eukaryotes are not apparent in the *D. discoideum* genome including ATM (Block and Lees-Miller, 2005; Hudson et al., 2005), a damage-activated protein kinase whose mutation in humans leads to Ataxia Telangiectasia. Similarly, *D. discoideum* undergoes a sexual life cycle with evidence for high levels of meiotic recombination but is missing an ortholog of Spo11, an enzyme required to initiate crossover during meiosis in other eukaryotes (Bloomfield, 2019; Bloomfield et al., 2019). Characterizing the molecular mechanisms which replace these “missing” components will reveal alternative pathways that might be exploited to ameliorate human diseases caused by their loss.

The majority of investigations into the mammalian DDR take place in proliferating cultured cells, which precludes an understanding of the effect of DNA damage and repair in a developmental context. Although unicellular when feeding, on starvation *D. discoideum* amoebae aggregate to form a multicellular structure which undergoes a rapid developmental process to generate a fruiting body with spores supported by a stalk comprised of dead, vacuolated cells (Kessin, 2001; Pears and Gross, 2021). This unusual developmental life cycle provides a tractable system in which to investigate any changes in repair activity during multicellular development and in differentiated cells (Kadam et al., 2021). It also facilitates analysis of the developmental consequences for cells accumulating DNA damage, as single cells can be fluorescently labeled and imaged throughout the process. Single cell transcriptomics has revealed a population of growing *D. discoideum* cells with high levels of spontaneous DNA damage, identified by expression of high levels of transcripts encoding the *D. discoideum* ortholog of Rad51, a repair protein involved in HR (Miermont et al., 2019). Interestingly cells expressing high levels of Rad51 are able to enter development but are frequently lost from the multicellular structure and excluded from entering the spore differentiation pathway. Prespore cells undergo a round of division during mid development (Muramoto and Chubb, 2008), so this might be explained by a block or delay in cell cycle progression caused by DNA damage inducing arrest at a cell cycle checkpoint. Spores are very resistant to DNA damage and loss of a prespore-specific catalase gene leads to sensitivity to hydrogen peroxide, an agent that induces predominantly single strand breaks (Garcia et al., 2003). Germinating spores have been shown to have an increased dependency on NHEJ to repair DNA DSBs as opposed to growing cells, which preferentially use accurate HR repair (Hudson et al., 2005). Understanding the interplay between development and DNA repair as well as the consequences of accumulation of mutations in differentiated tissue, will inform on the developmental consequences of inherited mutations in repair pathways, as seen in FA patients or neurological defects associated with genome instability syndromes such as Ataxia Telangiectasia or Nijmegen breakage syndrome.

## Conclusion

Preserving genome integrity through repair of DNA damage is critical for human health. Defects in repair pathways lead to a variety of diseases such as neurodegeneration, immune-deficiencies, congenital abnormalities and malignancy. The presence of repair pathways and components in *D. discoideum* lacking in other model organisms has been instrumental in revealing interplay between repair pathways with implications for targeting these in treatment of these, notably in cancer. The integration of traditional genetic and biochemical approaches along with next generation sequencing of both genomes and RNA in single cells, along with the ease of investigating the developmental program, will facilitate future discoveries.

## Author Contributions

CP wrote the manuscript. NL and JB revised and edited the manuscript. All authors contributed to manuscript revision, read, and approved the submitted version.

## Conflict of Interest

The authors declare that the research was conducted in the absence of any commercial or financial relationships that could be construed as a potential conflict of interest.

## Publisher’s Note

All claims expressed in this article are solely those of the authors and do not necessarily represent those of their affiliated organizations, or those of the publisher, the editors and the reviewers. Any product that may be evaluated in this article, or claim that may be made by its manufacturer, is not guaranteed or endorsed by the publisher.
